# Backbone and partial side chain assignment of the microtubule binding domain of the MAP1B light chain

**DOI:** 10.1007/s12104-013-9466-6

**Published:** 2013-01-22

**Authors:** Zsuzsanna Orbán-Németh, Morkos A. Henen, Leonhard Geist, Szymon Żerko, Saurabh Saxena, Jan Stanek, Wiktor Koźmiński, Friedrich Propst, Robert Konrat

**Affiliations:** 1 Department of Biochemistry and Cell Biology, Max F. Perutz Laboratories, University of Vienna, Vienna Biocenter Campus 1, 1030 Vienna, Austria; 2 Department of Structural and Computational Biology, Max F. Perutz Laboratories, University of Vienna, Vienna Biocenter Campus 5, 1030 Vienna, Austria; 3 Faculty of Chemistry, University of Warsaw, Pasteura 1, 02-093 Warsaw, Poland

**Keywords:** Microtubule-associated protein, Microtubule binding domain, Random sampling, Intrinsically disordered protein

## Abstract

Microtubule-associated protein 1B (MAP1B) is a classical high molecular mass microtubule-associated protein expressed at high levels in the brain. It confers specific properties to neuronal microtubules and is essential for neuronal differentiation, brain development and synapse maturation. Misexpression of the protein contributes to the development of brain disorders in humans. However, despite numerous reports demonstrating the importance of MAP1B in regulation of the neuronal cytoskeleton during neurite extension and axon guidance, its mechanism of action is still elusive. Here we focus on the intrinsically disordered microtubule binding domain of the light chain of MAP1B. In order to obtain more detailed structural information about this domain we assigned NMR chemical shifts of backbone and aliphatic side chain atoms.

## Biological context

During development and repair of the nervous system, individual neurons extend long processes to connect to each other to build or restore a network capable of information processing. Elaboration of these processes, axons and dendrites, crucially depends on microtubules, one component of the neuronal cytoskeleton. The dynamic behavior, the stability and the properties of microtubules are temporarily and spatially regulated by a plethora of microtubule-associated proteins (MAPs). Among these are the 3 members of the MAP1 family of proteins, MAP1A, MAP1B and MAP1S. All three are protein complexes consisting of heavy and light chains and share isolated domains of sequence homology (Schoenfeld et al. 1989; Orban-Nemeth et al. 2005).

The best characterized of these proteins is MAP1B, a large neuronal protein essential for neuronal network formation during murine brain development (Edelmann et al. 1996; Takei et al. 1997; Gonzalez-Billault et al. 2000; Meixner et al. 2000) and for the maturation of synapses (Tortosa et al. 2011). Overexpression or deregulation of MAP1B activity have been associated with fragile X mental retardation (Brown et al. 2001; Zhang et al. 2001), giant axonal neuropathy (Allen et al. 2005), and ataxia (Opal et al. 2003) in humans.

The MAP1B protein complex consists of a 300-kDa heavy chain and at least one light chain termed LC1. The light chain is around 250 amino acids (32 kDa) in size and has several important functional domains. Via its NH_2_-terminal domain it is capable of interacting with and changing the conformation of microtubules (Tögel et al. 1998). The light chains of MAP1A and MAP1S contain a functionally related microtubule binding domain in their respective NH_2_ termini; however, there is no sequence homology. In contrast, the COOH-terminal half of the MAP1B light chain is conserved in function as well as sequence among all MAP1 proteins. Via this part of the molecule the light chains can interact with actin filaments and with the heavy chains for the formation of the respective MAP1 protein complex (Tögel et al. 1998; Noiges et al. 2002, 2006; Orban-Nemeth et al. 2005). The interaction of the MAP1B light chain with microtubules can be regulated by posttranslational modification, in particular S-nitrosylation, at an identified cysteine residue in its COOH terminus (Stroissnigg et al. 2007). Being able to bind to microtubules as well as F-actin, MAP1B is considered to be a coupling protein between the two components of the neuronal cytoskeleton (Bouquet et al. 2007). Such proteins are postulated to be essential for orchestrated regulation of the cytoskeleton during neuronal differentiation (Lowery and Van Vactor 2009). However, despite the fact that MAP1B has been discovered many years ago and despite its demonstrated involvement in human disease a clear understanding of its function is still missing.

Meta structure analysis of the MAP1B light chain suggests that the positively charged NH_2_-terminal microtubule binding domain is intrinsically disordered, whereas the conserved COOH terminus is predicted to be structured. To obtain further insight into how this protein can modulate microtubule properties we initiated NMR structure determination of the NH_2_-terminal microtubule binding domain. This analysis might eventually also shed light on specific functions of microtubules during neuronal differentiation. Here we report near complete backbone assignment as well as partial assignment of aliphatic side chain atoms, using sophisticated 5D triple resonance NMR experiments.

## Methods and experiments

### Expression and purification of the NH_2_ terminus of the MAP1B light chain

A cDNA fragment encoding the NH_2_-terminal domain of the rat MAP1B light chain (amino acids 2212–2338, NP_062090.1) was cloned into a pET-15b (Novagen) derived expression vector. The resulting plasmid, pMA25His, encodes the microtubule binding domain of the MAP1B light chain fused to an NH_2_-terminal tag containing 6 histidines (MGSSHHHHHHSSGLVPRGSHMEF). The sequence was confirmed to be correct. pMA25His plasmid was introduced into BL21Codon Plus(DE3) cells (Stratagene) by heat shock at 42 °C for 90 s. Cells were grown overnight in LB medium at 37 °C supplemented with 50 μg/ml ampicillin and 25 μg/ml chloramphenicol. This culture was diluted 1:40 in 2 l LB with antibiotics and was incubated at 37 °C until the culture reached an OD_600_ of 0.7. Cells were collected at 5,000 rpm for 12 min and resuspended in half the volume of modified M9 minimal medium (Marley et al. 2001) supplemented with 1 g of ^15^NH_4_Cl and 2 g of D-[^13^C]glucose (Cambridge Isotope Laboratories) and trace elements instead of basal vitamins. Cells were incubated for one additional hour at 37 °C. Recombinant protein synthesis was induced by the addition of isopropyl-β-d-thiogalactopyranoside (IPTG) to a final concentration of 0.5 mM, and incubation was continued overnight at 18 °C. The cells were collected by centrifugation at 4,000 rpm for 15 min and resuspended in 10 ml of ice-cold lysis buffer (50 mM NaH_2_PO_4_, 300 mM NaCl, pH 8). Cells were lysed by sonication at 80 % for 20 × 10 s in 10 ml lysis buffer containing 40 mg lysozyme, 1 mg DNase I, and 4 EDTA free complete mini protease inhibitor tablets (Roche). The lysate was cleared by centrifugation at 13,000 rpm for 30 min. The supernatant was loaded onto an FPLC Ni^2+^-loaded HisTrap FF crude 5 ml affinity column (GE Healthcare). The column was washed with lysis buffer containing 20 mM imidazole. The recombinant protein was eluted with lysis buffer containing 75 mM imidazole, concentrated approximately 10-fold by centrifugation through an Amicon Ultra-15 centrifugal 3 K filter device and loaded onto a Superdex 200 HiLoad 16/60 prep grade gel filtration column (GE Healthcare) equilibrated in lysis buffer. The final yield of homogenous recombinant protein was approximately 2 mg/l of bacterial culture. For NMR analysis, protein samples were concentrated to at least 700 μM and pH was changed to 5.

### NMR experiments

All spectra were acquired at 298 K on an Agilent Direct Drive 700 MHz spectrometer using the standard 5 mm ^1^H–^13^C–^15^N triple-resonance probehead.

The backbone ^1^H, ^13^C and ^15^N resonances were assigned using sparse random sampling of indirectly detected time domains, in order to increase resolution. A 3D HNCO experiment was used as a base spectrum for SMFT (Sparse Multidimensional Fourier Transform) processing of higher dimensionality experiments (Kazimierczuk et al. 2009). Backbone assignment was achieved using 5D HN(CA)CONH (Kazimierczuk et al. 2010), (HACA)CON(CA)CONH (Zawadzka-Kazimierczuk et al. 2012b), (H)NCO(NCA)CONH (Zawadzka-Kazimierczuk et al. 2012b) and 4D HNCACO (Zawadzka-Kazimierczuk et al. 2010) experiments. Side-chain assignments were obtained using 5D HabCabCONH (Kazimierczuk et al. 2010), and H(CC-tocsy)CONH (Kazimierczuk et al. 2009) experiments.

All NMR data sets were processed by multidimensional Fourier transformation using the home written software package (http://nmr700.chem.uw.edu.pl/formularz.html). The resonance assignment was performed using the TSAR program (Zawadzka-Kazimierczuk et al. 2012a). The input data for TSAR was prepared and analyzed using the Sparky software (Goddard and Kneller 2008). Table 1 shows the maximum evolution times and spectral width used for the acquisition of the spectra.

**Table 1:** Maximum evolution times (tmax, ms) and spectral width (sw, kHz) used for acquisition of spectra for the NH_2_ terminus of the light chain of MAP1B

	3D HNCO	4D HNCACO	5D HabCabCONH	5D HN(CA)CONH	5D H(CC-tocsy)CONH	5D (H)NCO(NCA)CONH	5D (HACA)CON(CA)CONH
Number of points	750	1800	715	780	1285	700	900
Experiment duration (h)	5	23	18	20	42	18	19
sw_1_	2.8	2.8	4	6	8	2.5	3.8
sw_2_	2.5	6.2	14	2.5	18	2.8	2.8
sw_3_		2.5	2.8	2.8	2.8	2.8	2.8
sw_4_			2.5	2.5	2.5	2.5	2.5
*t*_1_^max^	100	50	15	20	15	30	30
*t*_2_^max^	100	10	7	50	8	30	30
*t*_3_^max^		75	30	50	30	30	30
*t*_4_^max^			50	50	50	50	50
Sampling density versus conventional	1.1 × 10^−2^	1.1 × 10^−3^	1.2 × 10^−5^	3.0 × 10^−6^	7.1 × 10^−6^	1.1 × 10^−5^	9.0 × 10^−6^

## Assignments and data deposition

**Fig. 1:**
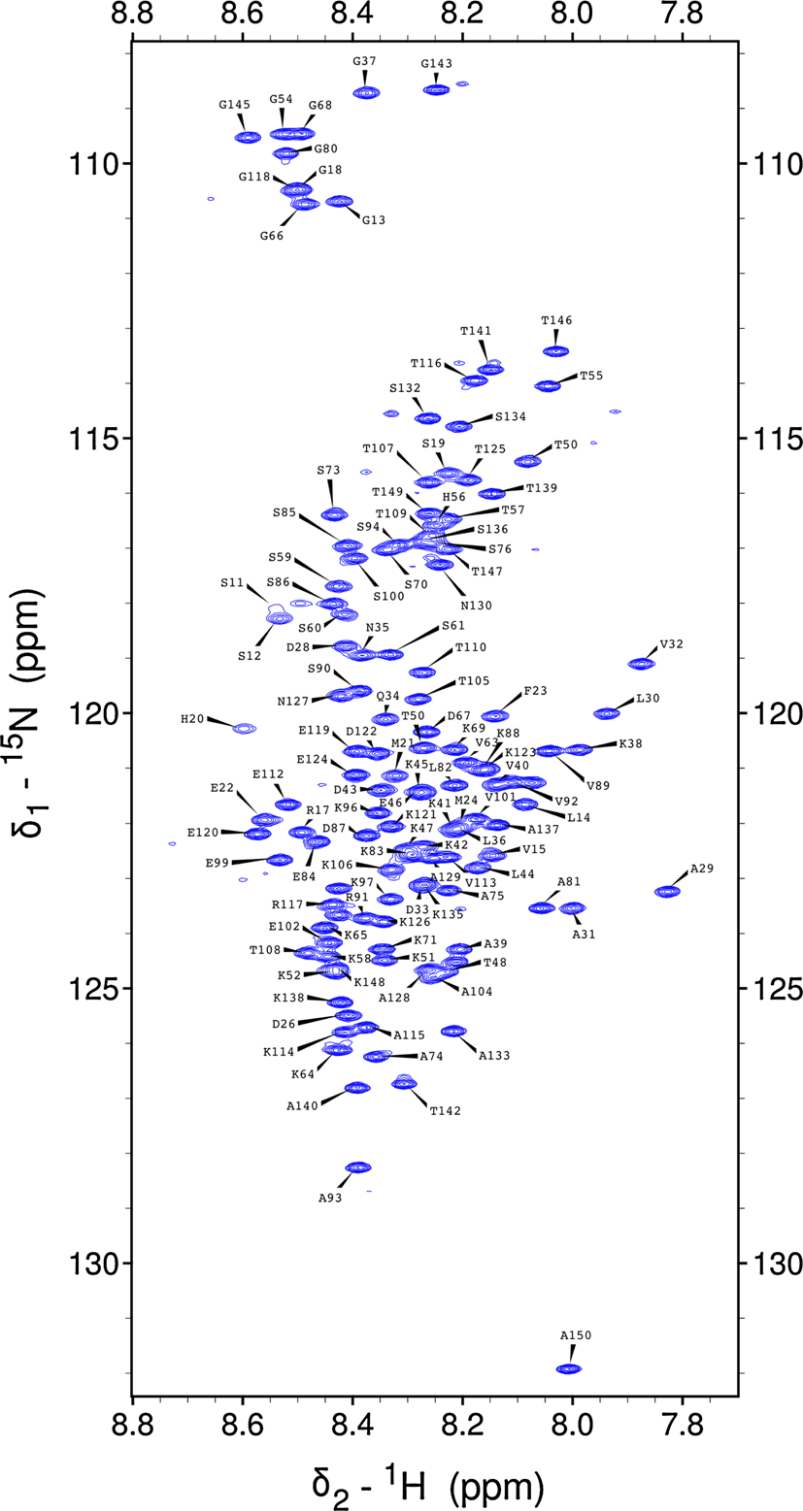
^1^H–^15^N HSQC spectrum of the NH_2_ terminus of the light chain of MAP1B at pH5 and 298 K. Assignments of backbone amides are labeled in *single letter* amino acid code and residue number (His6-tag: 1–23; NH_2_ terminus of the light chain of MAP1B: 24–150)

**Fig. 2:**
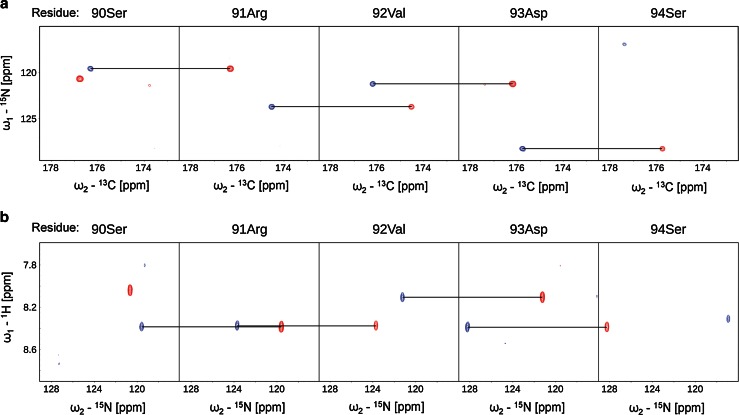
2D spectral planes for consecutive amino acids in the NH_2_ terminus of the light chain of MAP1B obtained by SMFT processing of the 5D randomly sampled signal. 2D cross-sections of **a** 5D (H)NCO(NCA)CONH (N_i_–CO_i−1_ and N_i−1_–CO_i−2_) and **b** 5D HN(CA)CONH (HN_i_–N_i_ and HN_i+1_–N_i+1_)

**Fig. 3:**
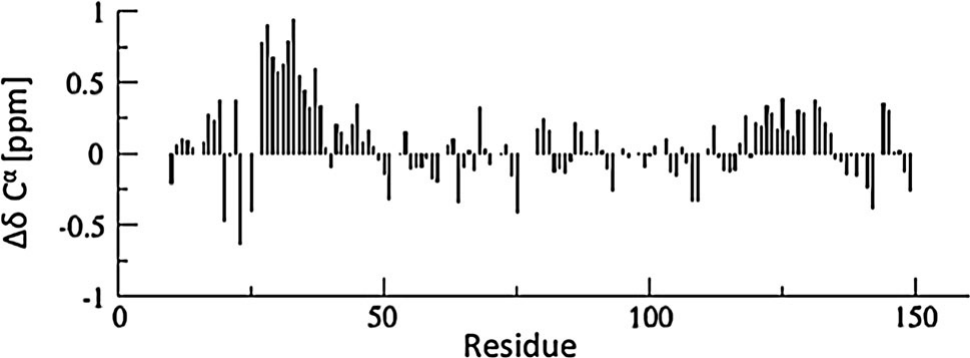
*Graph* showing ^13^C^α^ secondary chemical shifts of the NH_2_ terminus of the light chain of MAP1B. Random coil chemical shift values were obtained using the neighborhood-corrected IDP chemical shift library (Tamiola et al. 2010)

The ^1^H–^15^N HSQC spectrum of the NH_2_ terminus of the light chain of MAP1B shows the for intrinsically disordered proteins typical narrow peak dispersion in the ^1^H dimension (Fig. 1). The use of the aforementioned 5D experiments allowed us to nearly completely assign backbone atoms by resolving extensively overlapping signals in conventional 2D and 3D spectra. 89 % of backbone ^15^N, 95.8 % of ^1^H^N^, 88.2 % of ^13^C^α^, 88.2 % of ^1^H^α^ and 88.2 % of ^13^C^O^ resonances could be assigned (calculated without His-tag). Additionally, HabCabCONH and H(CC-tocsy)CONH spectra allowed the assignment of several aliphatic side chain atoms. 87.4 % of ^13^C^β^, 87.4 % of ^1^H^β^, 79.2 % of ^13^C^γ^, 85.2 % of ^1^H^γ^, 51.6 % ^13^C^δ^ and 62.7 % of ^1^H^δ^ could be assigned. For 7 out of 29 lysine side chains we also obtained the ^13^C^ε^ and ^1^H^ε^ assignments. Figure 2 outlines sequential resonance assignment in a 5D (H)NCO(NCA)CONH and HN(CA)CONH experiment, showing strips of sequential residues. Secondary chemical shifts for ^13^C^α^ (Fig. 3) show only minor deviations from random coil chemical shift values with slight α-helical propensities at the NH_2_ terminus, corroborating the finding of the Meta Structure analysis (not shown).

The ^1^H, ^13^C and ^15^N chemical shift assignments have been deposited in the BioMagResBank database (http://www.bmrb.wisc.edu) under the accession number 18895.
